# Congenital hypermetabolism and uncoupled oxidative phosphorylation

**DOI:** 10.1056/NEJMoa2202949

**Published:** 2022-10-13

**Authors:** Rebecca D. Ganetzky, Andrew L. Markhard, Irene Yee, Sheila Clever, Alan Cahill, Hardik Shah, Zenon Grabarek, Tsz-Leung To, Vamsi K. Mootha

**Affiliations:** 1 Mitochondrial Medicine Frontier Program, Division of Human Genetics, Children’s Hospital of Philadelphia, Philadelphia, PA 19104; 2 Department of Pediatrics, University of Pennsylvania Perelman School of Medicine, Philadelphia, PA 19104; 3 Howard Hughes Medical Institute and Department of Molecular Biology, Massachusetts General Hospital, Boston, MA 02114; 4 Metabolism Program, Broad Institute, Cambridge, MA 02142

## Abstract

We describe the case of identical twin boys who presented with low body weight despite excessive caloric intake. An evaluation of their fibroblasts showed elevated oxygen consumption and decreased mitochondrial membrane potential. Exome analysis revealed a *de novo* heterozygous variant in *ATP5F1B*, which encodes the β subunit of mitochondrial ATP synthase (also called complex V). In yeast, mutations affecting the same region loosen coupling between the proton motive force and ATP synthesis, resulting in high rates of mitochondrial respiration. Expression of the mutant allele in human cell lines recapitulates this phenotype. These data support an autosomal dominant mitochondrial uncoupling syndrome with hypermetabolism.

## Introduction

**Figure 1.**
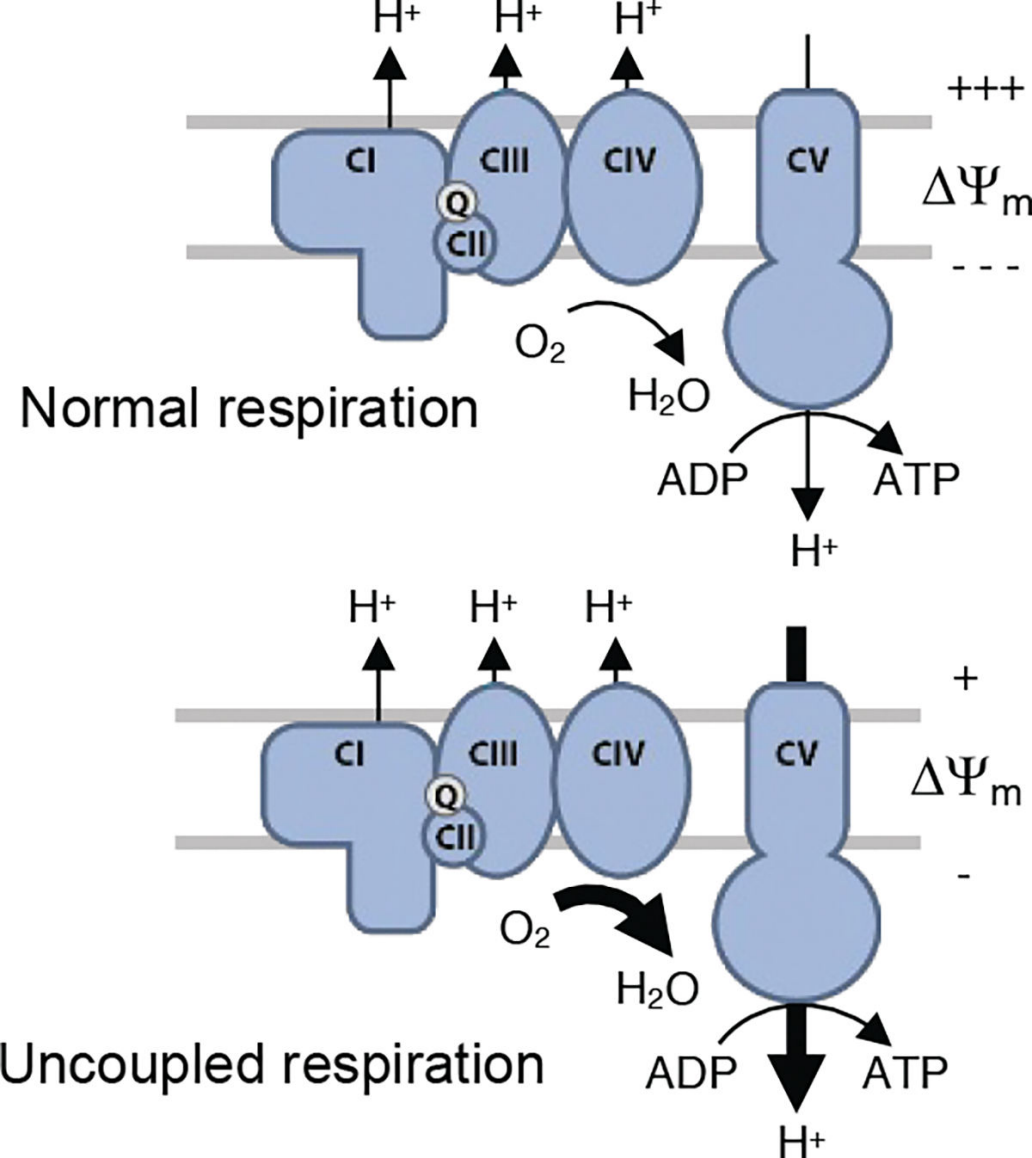
Oxidative phosphorylation in normal and variant cells. The oxidative phosphorylation (OXPHOS) pathway is a means of generating chemical energy, in the form of ATP, by oxidizing nutrients that are normally obtained from food. Electrons from nutrients are transferred by the respiratory chain complexes (CI-CIV) to oxygen, and in the process, energy is conserved in the form of a proton gradient, or proton motive force, across the mitochondrial inner membrane that consists primarily of a membrane potential. This proton gradient then drives efficient formation of ATP by complex V (CV). In healthy cells (top panel) the dissipation of the proton gradient is tightly coupled to the formation of ATP by complex V. The Leu335Pro variant in the ATP5B protein in twins A and B is predicted to loosen the coupling between the proton motive force and ATP synthesis by Complex V (bottom panel). As a consequence, these mitochondria are predicted to exhibit higher rates of respiration to defend the proton gradient for ATP production.

Mitochondrial oxidative phosphorylation (OXPHOS) is the major pathway for ATP production in eukaryotic cells Fig. 1. OXPHOS converts cellular redox energy (from the breakdown of food) to a proton gradient across the inner mitochondrial membrane (called the proton motive force), which is dissipated to catalyze the formation of adenosine triphosphate (ATP). Respiratory chain complexes I, III and IV pump protons across the inner mitochondrial membrane while consuming oxygen. Complex V (CV) is the central enzyme in energy conversion: it dissipates the proton gradient across the membrane, catalyzing the phosphorylation of ADP to ATP. In normally-functioning mitochondria, there is tight coupling between mitochondrial respiration and the formation of proton motive force, and between the dissipation of proton motive force and the synthesis of ATP. This tight chemiosmotic coupling between respiration and ATP synthesis ensures efficient energy transformations.

Over 300 molecular genetic forms of mitochondrial disease have been reported^1^. These disorders result from pathogenic variants in either the nuclear or mitochondrial DNA (mtDNA). Most are recessive conditions with decreased respiration. In contrast, the first described mitochondrial disease, Luft Syndrome, was a euthyroid, hypermetabolic state characterized by high rates of mitochondrial respiration “uncoupled” from ATP synthesis.^2,3^ Only one related case has been reported,^4,5,6^ but the molecular bases are unknown.

Two domains comprise CV: the transmembrane F_O_, which serves as a proton pore, and the catalytic F_1_.^7,8^ The catalytic core of F_1_ contains three α and three β subunits arranged around a γ subunit of the central stalk. Protons passing through F_O_ (the pore) cause the γ stalk to rotate, inducing conformational transitions in the β subunit that are required for the synthesis of ATP from ADP.^9,10^ The synthesis of ATP is thus dependent on (coupled to) the rotation of the γ stalk which, in turn, is dependent on (coupled to) the passage of protons through F_O_ (the pore); see Figure 1.

Here, we report the case of monozygotic twin boys who underwent their first biochemical genetics evaluation in the neonatal intensive care unit at the age of 2 months after presenting with a euthyroid hypermetabolism, failure to thrive despite excessive caloric intake, intermittent hyperthermia, and developmental delay. Genetic and biochemical studies in patient fibroblast and engineered cellular models implicate a dominant-acting, *de novo* pathogenic variant affecting CV that loosens coupling between the passage of protons (generated by mitochondrial respiration) through F_O_ and the synthesis of ATP by Complex V. (Complex V is also known as ATP synthase.)

## Methods

### Patient Phenotypes.

**Figure 2.**
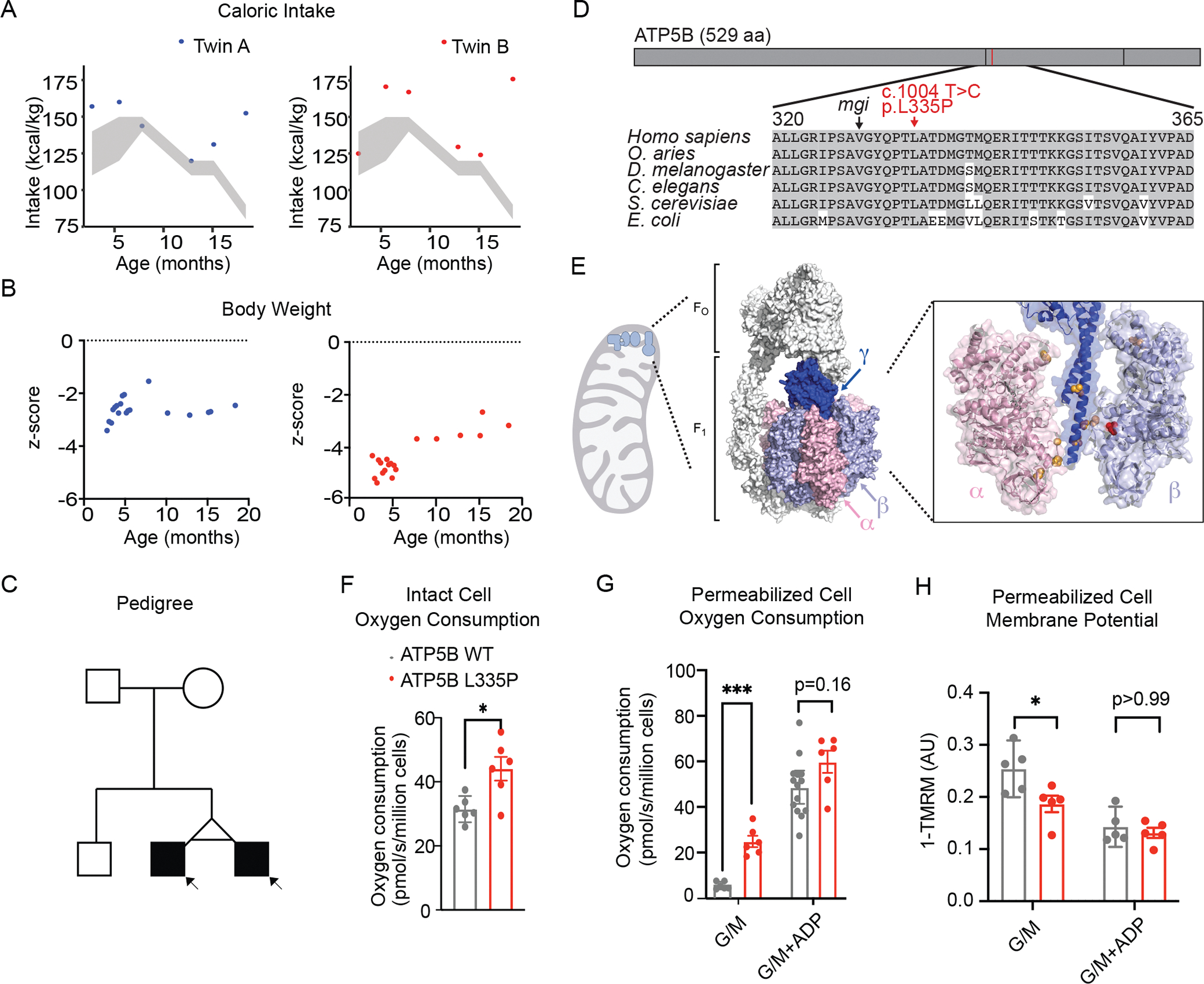
Newborn twins with evidence of hypermetabolism and uncoupled respiration. **A.** Caloric intake of Twin A (blue) and Twin B (red) at representative time points and the calculated caloric need for catch-up growth (shaded area). **B.** Weight of Twin A and Twin B calculated as Z-score based on the WHO weight for age standards. **C.** Pedigree of the reported family including the affected monozygotic twins. **D.** Multiple sequence alignment of the ATP5B protein indicating evolutionarily conserved residues (shaded), as well as the Leu335Pro mutation. Yeast *mgi* mutation sites are also denoted. **E.** Overview of the ovine CV structure (PDB: 6TT7)^12^ with a zoom into the patient mutation site (red sphere) in the beta subunit and the yeast *mgi* mutations (yellow spheres). **F.** Basal oxygen consumption in intact fibroblasts. **G.** Oxygen consymption in permeabilized fibroblasts in the presence of complex I substrates glutamate/malate (G/M) prior to addition of ADP and in the presence of saturating ADP. **H.** Change in polarization of the mitochondrial membrane in response to glutamate/malate (G/M), followed by ADP as represented by (1-TMRM intensity). Individual points represent n=6 (Fig. 1F), n=5 (Fig. 1G), n=5 (Fig. 1H) biological replicates. Means ± 95 % CI are shown.

Subjects 1 and 2 are monozygotic twins born at 34 weeks gestation with intrauterine growth restriction. Failure to thrive developed by 2 months of age and persisted despite caloric intake in excess of calculated need for catch-up growth. They had hyperphagia, with increasing caloric intake over time. At last follow-up (21 months), Twin A was consuming 1280 kcal/day (152 kcal/kg) and Twin B was consuming 1480 kcal/day (175 kcal/kg) (2.8 and 3.2 times the estimated resting energy expenditure Fig. 2A, respectively^11^). Their body-weight z-score was −2.5 and −3.2, respectively (Fig. 2B). They had persistent tachypnea (50–70 breaths/minute) without increased work of breathing, apnea, oxygen desaturations or pulmonary disease. Both had recurrent unexplained hyperthermia (38.0–39.0 °C). Baseline temperatures are 37.0–37.8 °C with persistent diaphoresis and tactile warmth. Subject 1 underwent chordee repair and intraoperatively developed an unexplained temperature of 40°C. Laboratory results were notable for elevated levels of branched-chain amino acids, mild hyperammonemia and uremia, and elevated triglycerides (Supplementary Table 1). They have mild global developmental delay (Supplementary Table 2). Their nonconsanguineous parents and their older brother were healthy (Fig. 2C).

### Creation of Primary Cell Line

Protocols for characterization of the patients and the creation of a primary cell line were developed in accordance with the requirements of the institutional review board at Children’s Hospital of Philadelphia. The twins’ parents provided written informed consent.

### Molecular and biochemical studies of cell lines.

Full methods are described in the Appendix. Briefly, primary dermoid fibroblast lines were established from the foreskin of each boy; a primary dermoid fibroblast line was used as control. CRISPR/Cas9 mediated knock-in clones of ATP5B Leu335Pro mutant and an isogeneic control were generated in A375 melanoma cells (Synthego Corporation). HeLa cells were engineered to heterologously express either ATP5B-FLAG or ATP5B-Leu335Pro-FLAG using lentiviral expression. For primary fibroblasts, oxygen consumption rate and mitochondrial membrane potential were measured simultaneously using an Oxygraph 2k fluorespirometer (Oroboros Instruments). Cells were permeabilized with digitonin and a substrate/uncoupler/inhibitor titration protocol was performed. For engineered A375 and HeLa cells, intact or permeabilized cell oxygen consumption rate and extracellular acidification (ECAR) rates were determined using a Seahorse XFe96 Analyzer (Agilent) with the standard oligomycin/carbonyl cyanide *m*-chlorophenyl hydrazone (CCCP)/piericidin+antimycin injection sequence. Mitochondrial membrane potential in intact cells was measured using tetramethylrhodamine methyl ester perchlorate (TMRM) with a BioTek Cytation5 (Agilent) plate reader. Sodium dodecyl sulphate-polyacrylamide gel electrophoresis (SDS-PAGE), Blue Native PAGE and western blotting were performed using standard methods employing commercially available antibodies.

### Structural Modeling

We used the cryo-EM structure of ovine CV (PDB: 6TT7)^12^ to model the Leu335Pro variant of human ATP5B. Positions of human Leu335Pro and yeast mitochondrial genome integrity (*mgi*) mutants were identified both by multiple sequence alignment and by structural alignment of the yeast (PDB:6CP6)^13^ and ovine CV. Structures were rendered with Pymol^14^.

### Statistical analysis.

**Figure 3.**
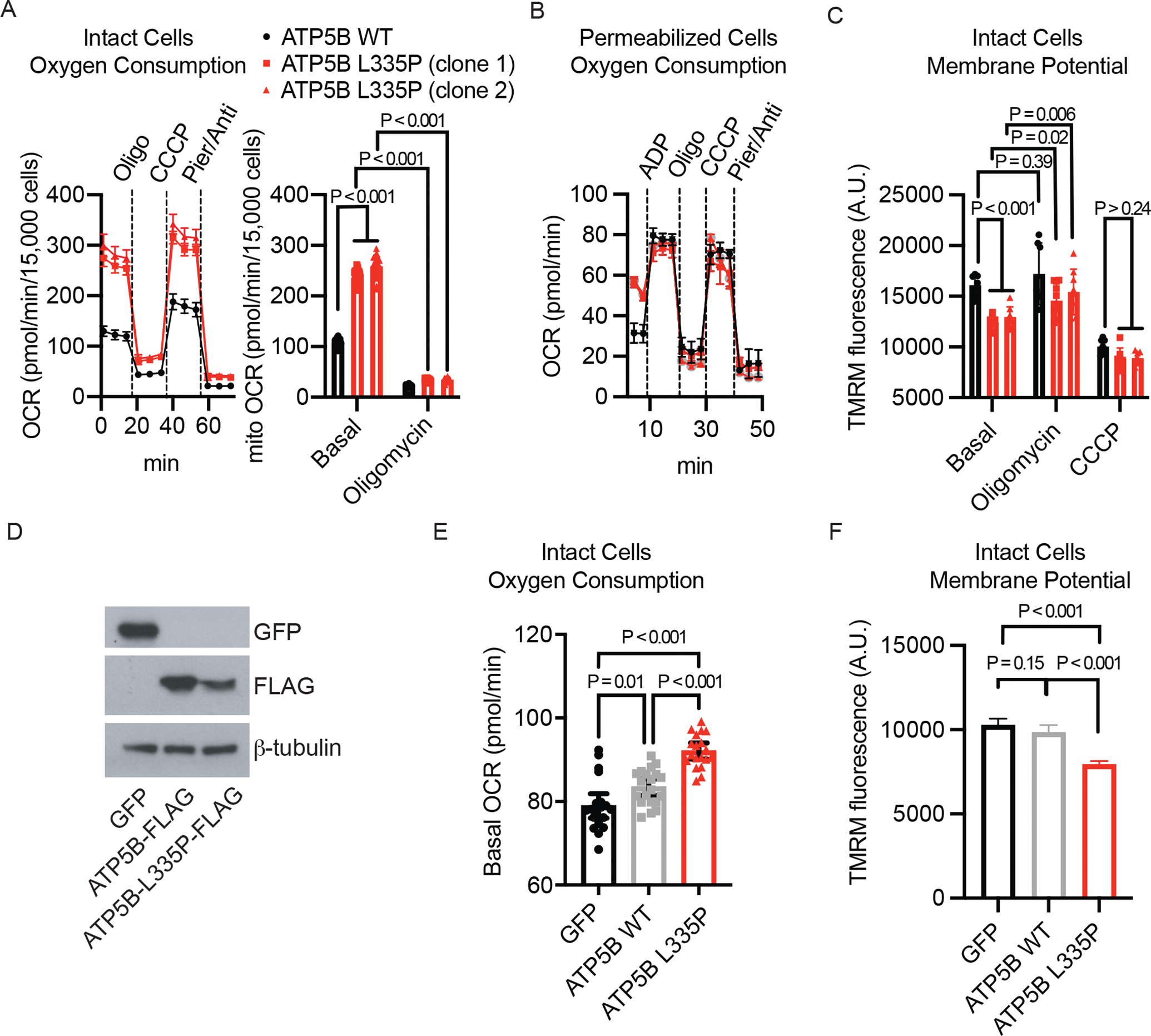
Bioenergetic characterization of engineered human cell lines. **A.** CRISPR was used to engineer the observed heterozygous mutation in isogenic A375 cell lines. The left panel shows Seahorse intact cell oxygen consumption rate measurements with sequential additions of oligomycin (CV inhibitor), CCCP (protonophore), and piericidin+antimycin (CI/CIII inhibitors). The right panel shows basal mitochondrial respiration and oligomycin-insensitive mitochondrial respiration estimated by subtracting the non-mitochondrial OCR (after piericidin+antimycin addition) from OCR at the baseline and OCR upon oligomycin addition, respectively. **B.** Seahorse permeabilized cell oxygen consumption rate measurements in the CRISPR engineered isogenic cell lines with sequential additions of ADP, oligomycin, CCCP, and piericidin+antimycin. **C.** TMRM intact cell membrane potential measurements in the CRISPR engineered isogenic cell lines under baseline, oligomycin, and CCCP conditions. **D.** HeLa cells were used for heterologous expression of the WT and Leu335Pro variant. Western blot analysis of GFP control, FLAG-tagged wild type ATP5B, or the FLAG-tagged Leu335Pro variant. **E.** Seahorse intact cell oxygen consumption measurements in the heterologous expression cell lines under basal condition. **F.** TMRM intact cell membrane potential measurements in the heterologous expression cell lines under baseline condition. OCR: oxygen consumption rate; A.U.: arbitrary units. Means ± standard deviations are shown. Individual points represent n=36 (Fig 3A), n=6 (Fig 3B), n=8 (Fig 3C), and n=18 (Fig 3E) biological replicates. Means ± 95 % CI of >300 single-cell measurements are shown for Fig 3F. In Panels A, C, and E, mean values for biologic replicates are shown; I bars indicate standard deviations. In Panel F, mean values for more than 300 single-cell measurements are shown; I bars indicate 95% confidence intervals. AU denotes arbitrary units.

We performed Mann–Whitney testing on the results of pulse oximetry (Fig. 2F and 2G) and Wilcoxon testing for TMRM measurements (Fig. 2H). We performed ordinary two-way analysis of variance comparing the mean value in each sample with that in every other sample, followed by Tukey’s multiple comparisons test, with a family-wise alpha threshold of 0.05 and with individual variances computed for each comparison (Fig. 3). We report P values for specified comparisons after adjustment for multiple comparisons. All the statistical analyses were performed with the use of RStudio software, version 1.1.456, and Prism software, version 9.

## Results

Exome analysis (Childen’s Hospital of Philadelphia) in the probands revealed an apparently *de novo* heterozygous variant NM_001686.3:c.1004 T>C in *ATP5F1B*, predicting a p.(Leu335Pro) change in the β subunit of CV. Both Leu335 and the surrounding region are highly conserved (Fig. 2D). Based on the gnomAD database^15^, *ATP5F1B* is highly constrained and the Leu335Pro variant has not been observed in any of ~200,000 individuals^15^.

Leu335 lies close to the hydrophobic sleeve in the α_3_β_3_ assembly that holds the tip of the γ subunit of the central stalk (Fig. 2E, S1). Mutations in this region in the α, β, or γ subunits in yeast are called *mgi* variants (Fig. 2E, S1) and result in uncoupling between ATP synthesis and mitochondrial respiration^13^. Leu335Pro is close to several *mgi* variants in the yeast equivalent of β.

We observed higher basal oxygen consumption rate in intact proband fibroblasts compared to control (Fig. 2F and Fig. S2). This difference was more pronounced in permeabilized cells following addition of the complex I-linked substrates glutamate/malate, but normalized following addition of saturating ADP (Fig. 2G and Fig. S2). In human mitochondria the membrane potential is the dominant component of the proton motive force. A cell line derived from Patient 2 showed comparatively diminished inner membrane polarization following addition of glutamate/malate, consistent with higher basal leak across the membrane (Fig. 2H and Fig. S2). Membrane potential following the addition of ADP was comparable to the control cell line. Overall, these results suggest the increased oxygen consumption rate is caused by dissipation of the proton motive force, without a corresponding increase in ATP synthesis – in other words, the dissipation of proton motive force has become less coupled from the synthesis of ATP. In such circumstances, the energy is lost in the form of heat, as opposed to being captured in the form of ATP.

To further corroborate these findings, we next used Cas9-gRNA (also known as CRISPR-Cas9) to engineer two independent clones with the heterozygous Leu335Pro mutation and compared them to an isogenic control (Fig. S3). As in the patient-derived fibroblasts, we observed elevated basal mitochondrial oxygen consumption rate in intact (Fig. 3A) and permeabilized mutant cells (Fig. 3B), associated with a lower baseline mitochondrial membrane potential (Fig. 3C). Both the increased oxygen consumption rate and lower mitochondrial membrane potential were normalized with oligomycin, which inhibits Complex V. Following treatment with a protonophore (which allows protons to move freely across the inner mitochondrial membrane) in permeabilized cells, oxygen consumption rate was similar between wildtype and mutant cells, corresponding to complete uncoupling and depolarization of the mitochondrial membrane in both. The oxygen consumption rate remained elevated in intact mutant cells, suggesting secondary changes in cytosolic metabolism. Indeed, we observed elevated baseline extracellular acidification rate, increased glucose consumption, and increased lactate release in the mutant cells, suggesting that secondary cellular adaptations include increased glycolysis (Fig. S4). Collectively, these studies in engineered, isogenic cell lines (Fig. 3A–C) support a mitochondrial uncoupling phenotype that biochemically originates from a variant affecting CV.

The Leu335Pro variant appears to have a dominant effect based on the familial pattern of inheritance. To distinguish between haploinsufficiency (insufficient wildtype β subunit) and dominant negativity (variant protein suppressing function of the wildtype protein), we heterologously expressed either an epitope-tagged wildtype (WT) or Leu335Pro variant in HeLa cells using lentiviral transduction. Cells tolerated expression of either allele and although the expression of the Leu335Pro variant was consistently lower than that of the WT (Fig. 3D), it assembled into macromolecular CV (Fig. S5A–B). Despite these lower levels of expression, baseline oxygen consumption rate (Fig. 3E) and glycolysis (Fig. S5C) were higher, and basal membrane potential was lower when compared with WT cells (Fig. 3F). Although in yeast, *mgi* mutations can cause mtDNA depletion, we did not observe changes in mtDNA copy number in our cell lines (Fig. S5D). The data support that heterologous expression of the Leu335Pro variant is sufficient to decrease basal mitochondrial membrane potential and increase respiration, consistent with a dominant-negative effect of the mutation on coupling.

Collectively, our analyses demonstrate the pathogenicity of the Leu335Pro variant. It is a confirmed *de novo* variant in the affected twin boys, absent from controls and affects an amino acid residue that is evolutionarily conserved. Leu335 is in the critical hydrophobic sleeve region; mutations in yeast affecting nearby residues cause an uncoupling phenotype. Oxygen consumption and membrane potential studies in both patient fibroblasts and cells with genetic introduction of the Leu335 variant show loosened coupling between the proton motive force (generated by mitochondrial respiration) and ATP synthesis due to intrinsic dysfunction of Complex V. Furthermore, heterologous expression of p.Leu335Pro on a wild-type background recapitulates these biochemical findings, consistent with a dominant-negative effect on chemiosmotic coupling. Based on these findings, the variant is classified as “pathogenic” by American College of Medical Genetics and Genomics criteria^16^ (ClinVar SCV002555570; Supplemental Appendix, Table S4).

## Discussion

Here we report monozygotic twin boys with euthyroid hypermetabolism characterized by excessive caloric intake, inability to gain weight, and tachypnea with a pathogenic variant (Leu335Pro) in *ATP5F1B* that results in a loosened coupling between dissipation of the proton motive force and the generation of ATP. At the time of this report, the twins continued to have persistent symptoms despite excess calorie provision, supplemental creatine to support muscle-energy metabolism, and supplemental folinic acid. Mitochondria in patient fibroblasts and in engineered heterozygous mutant cells have decreased mitochondrial membrane potential, presumably resulting from a greater flux of protons through CV and less efficient ATP production (Fig. 1). They also have increased oxygen consumption.

CV couples the proton motive force generated by the respiratory chain to the synthesis of ATP. The catalytic F_1_ domain of CV is comprised of a trimer of α–β dimers and a central γδε stalk^17^. The nucleotide-binding sites are located at the interface between each α and β subunit. Protons pass through the pore of F_O_, which is connected to the central stalk, driving its rotation within the α_3_β_3_ assembly. This rotation alters the affinity of the α–β interface for nucleotides, driving the catalytic cycle for ATP formation. Normally, every 360° rotation 8 protons are translocated into the matrix and 3 molecules of ATP are produced. Tight contacts between the γ subunit and the α_3_β_3_ assembly maximize efficient “powering” of ATP production by the translocation of protons.^17^ Importantly, the C-terminal tip of the γ subunit fits snugly in a hydrophobic sleeve made of amino acids 287–294 of the α subunits and amino acids 274–281 of the β subunits.^17^.

Our measurements of oxygen consumption and membrane potential show that if we chemically eliminate CV activity or, alternatively activate it maximally (saturating ADP), there are no residual differences in “mitochondrial leak,” demonstrating that uncoupling arises from a defect intrinsic to CV. Arsenieva and colleagues^13^ identified two categories of uncoupling *mgi* mutations in yeast CV. Group 1 mutations affect amino-acids that make up the hydrophobic sleeve of the α–β hexamer and allow the γ subunit to rotate without tightly engaging the α–β regions. Group 2 mutations alter the matrix-facing interface of the α and β subunits. Given the proximity of Leu335 to the hydrophobic sleeve and effect of certain yeast group 1 mutations, we propose that Leu335Pro is a group 1 mutation, because it probably impairs engagement of the γ subunit by the α–β hexamer by disrupting contact between the β and γ subunits. It thereby disrupts ATP synthesis, even with normal translocation of protons through F_O_.

We propose that the current condition and Luft Syndrome belong to a category of “mitochondrial uncoupling syndromes” characterized by elevated mitochondrial respiration uncoupled from mitochondrial ATP synthesis. Biochemically, however, Luft syndrome appears to be due to uncoupled respiration that originates outside of CV.^3^ Both conditions are characterized by high caloric intake, low body mass and recurrent hyperthermia.

Distinct clinical features in the twin brothers are presentation in infancy, developmental delay and episodic hyperthermia,^4^ the latter of which is consistent with the observation that uncoupling respiration from ATP synthesis results in heat generation in a variety of systems.^18,19^ While it is reasonable to assume that the hypermetabolism and hyperthermia are directly attributable to the effect of Leu335Pro on CV, it remains unclear why hyperthermia is episodic. Future challenges lie in delineating how the uncoupling defect within CV leads to secondary changes within mitochondria, the cell, and the patient.

We anticipate that mitochondrial uncoupling syndromes may have diverse molecular and biochemical etiologies. For example, uncoupling may be mediated by genetic variants that make the mitochondrial inner membrane leaky to protons. Alternatively, variants in the genes encoding the α, β, or γ subunits of CV may loosen coupling between the proton motive force and ATP synthesis ^13,20^. In the future, it will be interesting to determine whether inherited variation in these pathways may contribute to differences in energy metabolism in the broader population.

## Supplementary Material

Supplement
