# Supplementary material for: Congenital hypermetabolism and uncoupled oxidative phosphorylation
Source: N Engl J Med. Author manuscript; Available in PMC 2022 Dec 15. (PMC9754853; doi:10.1056/NEJMoa2202949)
Supplement: Supplement [file NIHMS1844632-supplement-Supplement.pdf]

Supplementary Appendix

Table of Contents

Investigators 2

Supplementary Methods 2

Figure S1. Additional renderings of the ovine CV structure depicting the Leu335pro mutation. 9

Figure S2. Representative oxygen and TMRM traces for patient fibroblasts. 10

Figure S3: Sequence confirmation of CRISPR engineered isogenic A375 cell lines. 11

Figure S4: Glycolytic phenotype of the CRISPR engineered isogenic A375 cell lines. 12

Figure S5: Additional analyses of heterologous expression of the WT and Leu335Pro variant in HeLa cell lines. 13

Table S1: Metabolic laboratories 14

Table S2: Developmental milestones achieved at 22 months of age 15

Table S3: cDNA, primer, and guide RNA sequences 16

Table S4: Classification of pathogenicity by American College of Medical Genetics and Genomics/Association of Molecular Pathology guidelines 21

References 22

Investigators: Rebecca D. Ganetzky MD, Andrew L. Markhard BA, Irene Yee, Sheila Clever MSc, Alan Cahill PhD, Hardik Shah MS, Zenon Grabarek PhD, Tsz-Leung To PhD & Vamsi K. Mootha MD

Supplementary Methods

Additional clinical description. Subjects 1 and 2 are of Ashkenazi Jewish ancestry born to a 34-year-old G3P1 mother. Estimated calorie intake for the first 6 months of life were based on strict intake recordings in the inpatient setting. Feeds were provided either directly by registered nurses or by parents under supervision by registered nurses. From 2-3 months of age parenteral nutrition was trialed to ensure absorption; however, this had no additional benefit. They were transitioned to fully enteral feeds at 3 months of age and by 15 months of age advanced to oral *ad lib* intake. From 6-15 months caloric calculations were based measured formula amounts provided by gastrostomy tube. After 15 months of age, caloric calculations were done based on three-day dietetic history provided by the parents that included measured portions. Average intake: average, 132 kcal/kg/day (Twin A); and Twin B consumed 134 kcal/kg/day; 130% of recommended daily caloric allowance and 110% of allowance recommended for catch-up.

Brain Magnetic Resonance Imaging (MRI) was performed and was normal. Mild laryngomalacia was diagnosed in both subjects. Continuous positive airway pressure was trialed for 3 days without change in respiratory rate; and they underwent supraglottoplasty without improvement of tachypnea or growth. There was no evidence of liver or renal dysfunction.

Quad exome included both twins, mother and father. The Leu335Pro variant was observed in neither parent. Genetic relatedness of the twins to both of their parents was confirmed

Engineering the endogenous locus of A375 cells to generate the heterozygous variant in ATP5F1B. Clustered Regularly Interspaced Short Pandemic Repeats (CRISPR)-Cas9 mediated knock-in clones of ATP5B Leu335Pro mutant and an isogeneic control carrying wild-type (WT) ATP5B were generated in A375 by Synthego Corporation (Redwood City, CA, USA). The designed short guide ribonucleic acid (sgRNA) and donor sequences are listed in Table S3. Silent mutations were included in the donor to modify the protospacer adjacent motif (PAM) site to reduce cutting post-editing and maximize the efficiency of the knock-in. To generate the knock-in cells, ribonucleoproteins containing the Cas9 protein and synthetic chemically modified sgRNA produced at Synthego were electroporated into the cells using Synthego's optimized protocol. Editing efficiency is assessed upon recovery, 48 h post electroporation. Genomic deoxyribonucleic acid (DNA) is extracted from a portion of the cells, polymerase chain reaction (PCR) amplified and sequenced using Sanger sequencing (Fig. S3). To create monoclonal cell populations, edited cell pools are seeded at <1 cell/well using a single cell printer into 96 or 384 well plates. All wells are imaged every 3 days to ensure expansion from a single-cell clone. Clonal populations are screened and identified using the PCR-Sanger-genotyping strategy.

Heterologous expression of the wild type and mutant ATP5F1B alleles in HeLa cells. ATP5B-FLAG and ATP5B-L335P-FLAG cDNA were custom synthesized in pUC57-Kan (GenScript; Supplementary Table 3). The complementary DNA (cDNA) was dissolved in TE Buffer (Invitrogen #12090-015), digested using NheI-HF and EcoRI-HF restriction enzymes (New England Biolabs #R3131, #R3101), gel extracted (Qiagen #28704), and ligated into the pLYS1

lentiviral expression vector (Addgene #50057) using Quick Ligase (New England Biolabs #M2200). Ligations were transformed in XL-10 Gold competent cells (Agilent #200315) and cells were grown on LB/Ampicillin plates overnight at 37°C. Single colonies were picked, grown in 5 ml LB/Ampicillin media overnight at 37°C, and miniprepped (Qiagen #27106). Construct sequences were verified by Sanger sequencing (Genewiz) using CMV-Forward and pLJM-reverse sequencing primers (Supplementary Table 3). To generate lentivirus, 10^6 293T cells (ATCC #CRL-3216) were seeded per plate in 6 cm culture dishes, in 5 ml media. The next day, the cells were transfected using X-tremeGENE HP transfection reagent (Roche #6366244001) with 1 mg of the pLYS1-ATP5B-FLAG or pLYS1-ATP5B-L335P-FLAG construct or a pLJM1-EGFP control lentiviral expression vector (Addgene #19319), along with 900 ng of psPAX2 (Addgene #12260) and 100 ng of pCMV-VSV-G (Addgene #8454) lentiviral packaging and envelope plasmids. Forty-eight h post transfection, lentivirus was collected and passed through a 0.45 mm polyethersulfone syringe filter (Whatman #6780-2504). $2x10^5$ HeLa cells (ATCC #CCL-2) were seeded per well in 6-well culture plates, in 2 ml media per well. The next day, the cells were treated with 8 mg/ml polybrene (Sigma #H9268) and infected with 300 ml lentivirus, with spinfection by centrifugation at 1000 x g for 30 min, at 37°C. After 48 h, cells were passaged and selected with 2 mg/ml puromycin (Gibco #A1113803). Cells were maintained in puromycin for an additional passage prior to use for subsequent experiments. 293T and HeLa cells were cultured in Dulbecco's Modified Eagle Medium (DMEM, Gibco #11995-065) supplemented with 10 % fetal bovine serum (FBS, Sigma #2442), 1X GlutaMax (Gibco #35050061), and penicillin/streptomycin (Gibco #15140122). Cells were maintained in a 37°C, 5% CO_2 incubator unless otherwise indicated.

Bioenergetics measurements in primary cells. For primary fibroblasts, oxygen consumption and mitochondrial membrane potential were measured simultaneously using an Oxygraph 2k fluorespirometer (Oroboros Instruments) using standard permeabilized cell protocols[1]. Control fibroblasts were age- and sex-matched, as well as matched for passage number. Prior to use, cells were cultured in low glucose DMEM (Gibco #11-885-092), supplemented with 10 % FBS and 10 µg/mL uridine and maintained in a 37°C, 5% carbon dioxide (CO_2) incubator. Cells were harvested at 85-90% confluence. In brief, 2.2 mL chambers filled with Mir05 media were used. For measurement of mitochondrial membrane potential, two stepwise additions of tetramethylrhodamine methyl ester perchlorate (TMRM; Molecular Probes) to a final concentration of 0.2 mM were added. A linear calibration curve of TMRM concentration to fluorescence detected was calculated from these additions. 10^6 cells were then added followed by 3.2 mmol malate. Cells were permeabilized with digitonin titrated to achieve oxygen consumption less than 15 pmol/s/million cells (final concentration approximately 100 mg/mL) . A substrate/uncoupler/inhibitor titration protocol (SUIT) was then performed with sequential additions of 40 mmol/L glutamate (Sigma), 2.5 mmol/L ADP (Sigma), 40 mmol/L succinate (Sigma), carbonyl cyanide p-trifluoro-methoxyphenyl hydrazone (FCCP; Sigma) in 0.5 mmol/L titrations until maximum oxygen consumption was reached, 2 nmol/L Rotenone (VWR) and 5 nmol/L Antimycin A (Sigma). The residual non-mitochondrial oxygen consumption (following addition of rotenone and antimycin A) was subtracted from all other oxygen consumption rates. TMRM signal quenching is measured, therefore depolarization is inferred from increased intensity and hyperpolarization from decreased intensity.

Bioenergetics measurements in A375 and HeLa cells. For both A375 and HeLa cells, intact cell oxygen consumption and extracellular acidification rates (OCR and ECAR) were determined using a Seahorse XFe96 Analyzer (Agilent). For OCR and ECAR measurements in A375 cells, cells were seeded at $1.5x10^4$ cells in 100 ml/well in 96-well Seahorse cell culture plates, in DMEM supplemented with 10% FBS (Gibco # 26140-079), 1X GlutaMax (Gibco #35050-061), and pen/strep (Gibco #10378-016). After 14 h, 80 ml of media was removed and 160 ml of HEPES buffered Seahorse DMEM (Agilent #103575-100) supplemented with 25 mM glucose (Sigma #G8270), 1 mM sodium pyruvate (Gibco #11360-070), and 1X GlutaMax was added, and the plate was transferred to a 37°C non-CO_2 incubator for one h. The Seahorse cartridge was hydrated according to the manufacturer's protocol. Oligomycin A (Sigma), CCCP, and Piericidin A (Cayman Chemical) + Antimycin A (Sigma) were prepared in Seahorse DMEM and added to the cells at final concentrations of 2 μM (Oligomycin), 2 μM (CCCP) and 1 μM +1 μM (Piericidin+Antimycin), respectively. Three baseline respiratory rate measurements were taken, followed by sequential injections of each inhibitor with three measurements each (cycles of: 3 min mix, 0 min wait, 3 min measure). After the Seahorse run, 2 μg/mL Hoechst 33342 (Invitrogen #H3570) was added to each well and incubated for 10 min, and the plate was imaged on a BioTek Cytation 5 Cell Imaging Multi-Mode Reader (Agilent # CYT5MW) using a DAPI filter cube (Agilent #1225100). Stained nuclei were counted using the Gen5 Image Prime Software (Agilent #GEN5IPRIME) and the total number of nuclei in each well (a proxy for the cell number) was used to normalize the Seahorse OCR and ECAR measurements. The values of individual biological replicates at the first time points post treatment were shown in the right panel of Fig. 3A. Basal mitochondrial respiration and oligomycin-insensitive mitochondrial respiration were estimated by subtracting the non-mitochondrial OCR (after piericidin+antimycin addition, final time point) from OCR at the baseline and OCR upon oligomycin addition, respectively, for individual biological replicates.

For permeabilized Seahorse OCR measurements, A375 cells were seeded at $3x10^4$ cells/well in 80 μl/well growth media and grown overnight at 37C. Seahorse cartridges were hydrated overnight at 37C, according to the manufacturer's protocol. The next day, cells were washed once with MAS buffer (70 mM sucrose, 220 mM mannitol, 5 mM KH_2PO_4, 5 mM $MgCl_2$, 2 mM HEPES, 1 mM EGTA, 0.2% FA-free BSA). Cells were then permeabilized with MAS buffer supplemented with 2 nM XF Plasma Membrane Permeabilizer (Agilent 102504-100) and 5 mM each of glutamate and malate. Upon assay start, baseline respiratory rate measurements were taken (cycles of: 0.5 min mix, 0.5 min wait, 2 min measure), followed by injection of ADP (0.4 mM), oligomycin (2 μM), CCCP (8 μM) and Piericidin + Antimycin (1 μM each) and three respiratory rate measurements after each injection.

For OCR and ECAR measurements in HeLa cells, cells were seeded at $1.5x10^4$ cells in 100 ml/well in 96-well Seahorse cell culture plates, in DMEM supplemented with 10% dialyzed FBS (dFBS, Gibco #26400-044), 1X GlutaMax (Gibco #35050-061), and penicillin/streptomycin (Gibco #10378-016). After 14 h, 80 ml of media was removed and 160 ml of HEPES buffered Seahorse DMEM (Agilent #103575-100) supplemented with 25 mM glucose (Sigma #G8270), 10% dFBS, 1 mM sodium pyruvate (Gibco #11360-070), and 1X GlutaMax was added, and the plate was transferred to a 37°C non-CO_2 incubator for one h. The Seahorse cartridge was hydrated according to the manufacturer's protocol. Oligomycin A (Sigma), FCCP, and Piericidin A (Cayman Chemical) + Antimycin A (Sigma) were prepared in Seahorse DMEM and added to

the cells at final concentrations of 2 μM (Oligomycin), 2 μM (FCCP) and 1 μM +1 μM (Piericidin+Antimycin), respectively. Four baseline respiratory rate measurements were taken, followed by sequential injections of each inhibitor with three measurements each (cycles of: 3 min mix, 0 min wait, 3 min measure). The values of individual biological replicates at the first time points post treatment were shown in Fig. 3E.

For membrane potential measurements in intact A375 cells, cells were seeded in 96 well glass bottom plates (Corning #3904) at $2x10^4$ cells in 100 ml/well in DMEM supplemented with 10% FBS and pen/strep (Gibco #10378-016). After 14 h, cells were treated with 50 nM TMRM (Invitrogen #I34361), and 0.1% dimethyl sulfoxide (DMSO; Invitrogen # D12345) or 1 μM carbonyl cyanide 3-chlorophenylhydrazone (Sigma # C2759) or 2 μM oligomycin A (Sigma Cat # 75351). Following additions of TMRM, and drugs, cells were incubated in a 37°C, 5% CO_2 incubator for 30 min. Media was removed and cells were washed with 100 ml of (4-(2-hydroxyethyl)-1-piperazineethanesulfonic acid) HEPES buffered Seahorse DMEM supplemented with 25 mM glucose, 2 mM L-glutamine, 1 mM sodium pyruvate, and imaged in this media. Fluorescence spectroscopy was performed on a BioTek Cytation 5 Cell Imaging Multi-Mode Reader (Agilent # CYT5MW). Fluorescence spectra were collected from the emission range of 560-620 nm using an excitation of 540 nm. The peak fluorescence values (at 585 nm) were used for comparison. For each condition, n=8 biological replicates were used.

For membrane potential measurements in intact HeLa cells, cells were seeded in 24 well glass bottom plates (Cellvis #P24-1.5H-N) at $9x10^4$ cells in 600 ml/well in DMEM supplemented with 10% FBS and pen/strep (Gibco #10378-016). After 14 h, cells were treated with 50 nM TMRM (Invitrogen #I34361) and 2 μg/mL Hoechst 33342 (Invitrogen #H3570). Following additions of TMRM, Hoechst, and drugs, cells were incubated in a 37°C, 5% CO_2 incubator for 30 min. Media was removed and cells were washed with 500 ml of HEPES buffered Seahorse DMEM supplemented with 25 mM glucose, 2 mM L-glutamine, 1 mM sodium pyruvate, and imaged in this media. Fluorescence imaging was performed on a BioTek Cytation 5 Cell Imaging Multi-Mode Reader (Agilent # CYT5MW), using a 20X objective (Agilent # 1220517). Hoechst signals were acquired using a 365 nm light emiting diode (Agilent #1225007) and a (4′,6-diamidino-2-phenylindole) DAPI filter cube (Agilent #1225100). TMRM signals were acquired using a 523 nm LED (Agilent #1225003) and a red fluorescent protein (RFP) filter cube (Agilent #1225103). Quantification of TMRM signals was performed using the Gen5 Image Prime Software (Agilent #GEN5IPRIME). Briefly, a primary cell mask was created for each cell using by thresholding with the Hoechst signal. This cell mask was expanded for 3 mm to include the cytoplasm. The TMRM signal for each cell is taken as the mean intensity within the cell mask. For each condition, the average intensity was calculated as the mean TMRM signal from all cells across multiple frames of view (> 300 cells in total).

Media glucose and lactic acid quantification
A375 cells carrying wild-type *ATP5F1B* or the heterozygous Leu335Pro mutation (2 individual clones) were seeded in 6-well plates overnight in DMEM media containing 10 % FBS, 25 mM glucose, 1 mM pyruvate, 2 mM L-glutamine, and 50 μg/ml uridine in 6-well plates. On the day of the experiment, cells (about $5x10^5$ per well) were incubated with 1.5 mL of fresh media in each well of 6-well plates for 8 h and control wells without cells were included to account for the evaporation effect. After 8 h of incubation, 200 μL of spent media sample was spun down to

remove any dead cells and the supernatant was stored at -80°C until analysis. Metabolite profiling of the spent media was performed according to a previously established protocol[2].

Native polyacrylamide gel electrophoresis (PAGE)
Protein complexes isolated from digitonin-solubilized mitochondria from patient fibroblasts were analyzed using the NativePAGE Novex Bis-Tris gel system. Briefly, mitochondrial pellets were solubilized on ice in Invitrogen NativePAGE sample buffer at 2.5 mg/mL using digitonin (8 g digitonin / g mitochondrial protein) for 20 min. Following clarification (13,000 x g, 10 min) to remove insoluble material, liberated protein complexes were supplemented with 5% Coomassie G-250 (final concentration 1/4th that of the digitonin concentration) and separated through Invitrogen 3-12% bis-tris NativePAGE gels using a hybrid version of the blue-native/clear-native procedures. Complexes were first run for 30 min at 150V using a cathode buffer (Invitrogen NativePAGE running buffer) supplemented with Coomassie G-250 (44 mg / 220 mL). The cathode buffer was then switched to a clear NativePAGE running buffer for a further 1h. NativePAGE running buffer was also used as the anode buffer. HeLa cell lines were grown to ~90% confluence in 15 cm culture dishes. Cells were washed with ice cold phosphobuffered saline (PBS). PBS was aspirated and 1 ml of ice cold IBc buffer (200 mM sucrose, 10 mM Tris-3-(*N*-morpholino)propanesulfonic acid (MOPS) pH 7.4, 1 mM ethylene glycol-diamine tetraacetic acid (EGTA)-Tris pH 7.4) supplemented with protease inhibitor (Roche #05056489001) was added, and cells were scraped and collected in a microcentrifuge tube on ice. Samples were centrifuged at 800 x g for 3 min at 4°C to pellet cells. Supernatant was carefully removed and cell pellet was resuspended in 900 ml of IBc buffer. Plasma membranes were mechanically disrupted by passing samples 15 times in and out of a 1 ml syringe through a 27 gauge x 1/2 needle, on ice. Cell lysates were centrifuged at 800 x g for 10 min at 4°C. Supernatants were carefully transferred to new microcentrifuge tubes, and samples were centrifuged at 8000 x g for 6 min at 4°C. Supernatant was carefully removed, and pellet was resuspended in 50 ml IBc buffer. One ml of crude mitochondria was diluted in 9 ml of radio immunoprecipitation assay (RIPA) buffer (Boston BioProducts #BP-115DG), and protein content was measured by BCA assay (Pierce #23227). Crude mitochondria were solubilized at 1 mg/ml in 1X NativePage Sample Buffer with 2% n-Dodecyl β-D-maltoside (DDM, Sigma #D4641), on ice. Samples were clarified by centrifugation at 20,000 x g for 30 min at 4°C. Supernatants were transferred to new microcentrifuge tubes, on ice. NativePAGE G-250 (Invitrogen #BN2004) was added to a concentration of 0.2%. Samples were run alongside NativeMark Unstained Protein Standard (Invitrogen #LC0725) on 3-12% 10-well 1.0 mm Native PAGE Bis-Tris gels (Invitrogen #BN1001BOX), at 4°C. Gel running buffers were prepared according to the manufacturer's protocol (Invitrogen). Gels were run at 150 volts for approximately 20 minutes with dark cathode buffer, until the dye front had migrated through one third of the gel. The dark cathode buffer was then replaced with light cathode buffer, and the gels were run at 250 volts for an additional 85 minutes.

Immunoblotting and fluorescence detection
For samples from primary fibroblasts, gels were transferred to Invitrogen polyvinylidene difluoride (PVDF) membranes (0.2 μm pore size) overnight at constant voltage (30V) using a BioRad Mini Protean transfer system with Invitrogen NuPage transfer buffer. Membranes were blocked with LI-COR Intercept TBS blocking buffer and probed with Abcam anti-ATP5A antibody (ab14748) and a LI-COR IRDye 680RD conjugated secondary antibody. Fluorescence

was detected using an Amersham Typhoon Biomolecular Imager (GE Healthcare) with a 685 nm excitation wavelength and an 720BP20 (IRshort) emission filter. For HeLa cell Western blots from sodium dodecyl sulfate (SDS)-PAGE, cells were washed with ice cold PBS, and lysed with ice cold RIPA buffer supplemented with protease/phosphatase inhibitor (Cell Signaling #5872). Lysates were clarified by centrifugation at 21,000 x g for 10 min, at 4°C. Supernatants were transferred to new microcentrifuge tubes on ice. Protein concentrations were quantified by BCA assay. Samples were normalized to 1 mg/ml in RIPA buffer with 1X SDS sample buffer (2% SDS, 5% β-mercaptoethanol, 5% glycerol, 47.4 mM Tris-HCl, 16.6 µM Bromophenol Blue, pH 6.8). Samples were heated for 5 minutes on a 95°C heat block and cooled at room temperature before loading on SDS-PAGE gels. Samples were run alongside Spectra Multicolor Broad Range Protein Ladder (Thermo Scientific #26623) on 12% or 4-20% 1.0 mm Tris-Glycine gels (Life Technologies #XP00125BOX, #XP04205BOX) at 120 volts for approximately 2 h, in 1X Tris-Glycine running buffer (Boston BioProducts #BP-150). Gels were transferred to PVDF membranes (0.2 µm pore size, Bio-Rad #1704157) using a Trans-Blot Turbo Transfer System (Bio-Rad) set to the mixed molecular weight program. Membranes were blocked with 5% non-fat dry milk (Bio-Rad #1706404XTU) in 1X Tris Buffered Saline-Tween (TBST, Boston BioProducts #IBB-580) for 1 h at room temperature. Membranes were probed with anti-GFP (Abcam #ab6556), anti-FLAG (Cell Signaling #2368), anti-β-tubulin (Cell Signaling #2128), or the Human Total OXPHOS antibody cocktail (Abcam #ab110411). All primary antibodies were used at 1:1000 dilution in 5% milk/TBST overnight at 4C. HRP-conjugated donkey anti-rabbit (Cell Signaling #7074) and sheep anti-mouse (GE Healthcare #NA931V) secondary antibodies were used at 1:5000 dilution in 5% milk/TBST for 1 h at room temperature. Membranes were washed 6 x 5 min with 1X TBST before and after secondary antibody incubation. Membranes were incubated with Western Lightning Plus electrochemiluminesence (ECL) substrate (PerkinElmer #NEL104001EA) for 3 min. Luminescence was detected using Amersham Hyperfilm ECL film (GE Healthcare #28906838) developed on an X-Omat 2000A Processor (Kodak).

For HeLa cell Western blots from Blue NativePAGE, gels were transferred to PVDF membranes (0.2 µm pore size, Invitrogen #LC2002) with 1X NuPAGE transfer buffer (25 mM Bicine, 25 mM Bis-Tris, 1.025 mM ethylene diamine tetra acetic acid (EDTA), pH 7.2) using a Bio-Rad Trans-Blot SD semi-dry transfer cell at 180 mA constant current for 20 minutes. After transfer, the molecular weight marker was cut from the rest of the membrane and stained with Imperial Protein Stain (Invitrogen #24615). The remainder of the membrane was incubated in 8% acetic acid for 5 min with gentle agitation to fix the proteins, then washed with ddH_2O for 5 min. The membrane was then placed in methanol and shaken for ~1 min. The methanol was refreshed and shaking continued for an additional 30 sec. The membrane was then washed with multiple changes of 0.5X TBST for 5 min, followed by blocking with 5% milk/TBST as described above. Membranes were probed with the Human Total OXPHOS antibody cocktail or anti-FLAG. All primary antibodies were used at 1:1000 dilution overnight at 4C. Secondary antibodies, ECL, and film detection were used as described above for HeLa cell Western blots. Membranes were stripped using Restore Stripping Buffer (Pierce #21059), blocked, and reprobed with anti-VDAC (Cell Signaling #4866).

Coomassie staining

For staining of protein complexes, gels were incubated in Abcam Instant Blue Coomassie protein stain overnight then washed in water before being scanned using the densitometry settings of the Amersham Typhoon Biomolecular Imager.

Mitochondrial DNA (mtDNA) copy number analysis
For assessment of mtDNA copy number, cells were trypsinized and counted. $2.5x10^6$ cells were collected in a 15 ml Falcon tube and pelleted by centrifugation at 800 x g for 3 min. The supernatant was aspirated. DNA was extracted from cells using the Qiagen Blood & Tissue DNeasy kit according to the manufacturer's protocol (Qiagen #69506). DNA was quantified on a NanoDrop OneC (Thermo Scientific). Matching pairs of forward and reverse qPCR primers were combined at 10 µM concentration (for primer sequences see Supplementary Table 3). Reaction mixes were prepared by combining 30 ng DNA, 1 µl of 10 µM primer mix, 10 µl of iQ SYBR Green Supermix (Bio-Rad #1708880), and UltraPure dH2O (Invitrogen #10977-015) to 20 µl total volume. qPCR assays were run on a CFX96 Real-Time System (Bio-Rad). qPCR parameters: 95°C for 3 min, 40 cycles of: 95 °C for 10 s + 60 °C for 30 s + plate read, melt curve ramp: 65 °C to 95°C in 0.5 °C increments for 5 s at each temperature + plate read. Mean Cq values were calculated from technical triplicate wells. ΔCq values were calculated between mtDNA and nuclear DNA (nDNA) primer pairs, with relative mtDNA/nDNA content reported as $2x2^{\Delta Cq}$ mean normalized to green fluorescent protein (GFP) control[3].

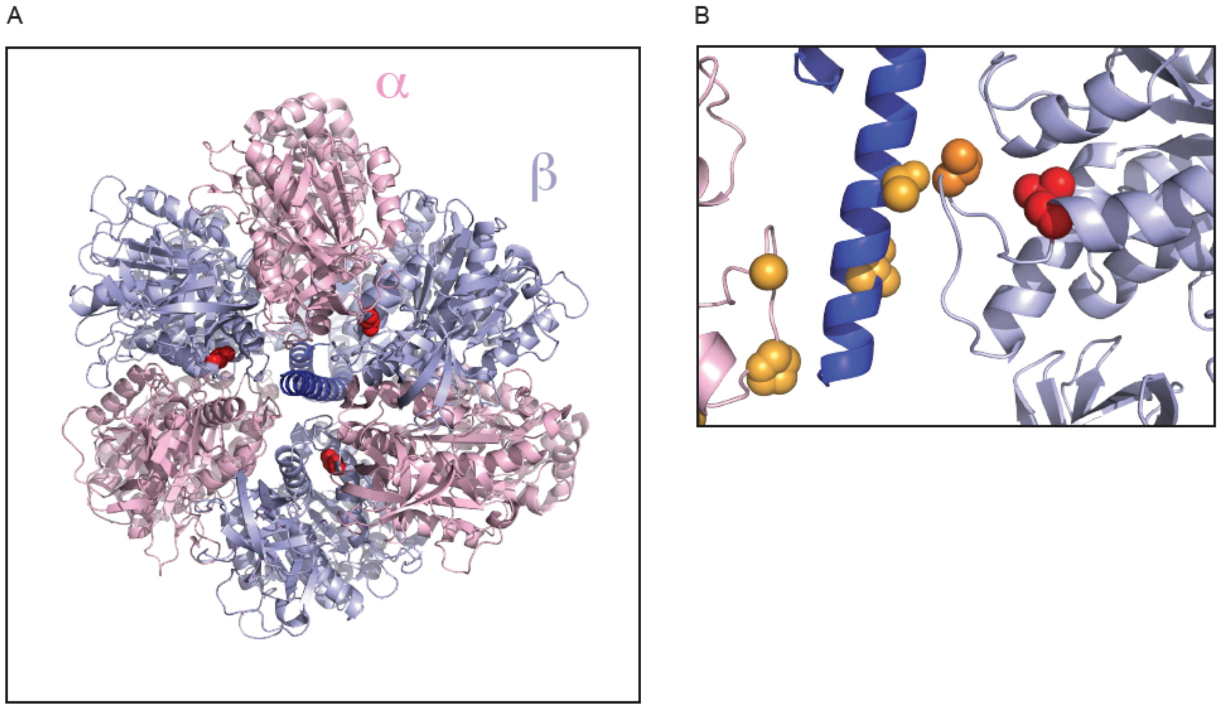

Figure S1. Additional renderings of the ovine CV structure depicting the Leu335pro mutation. A. View of the F_1 domain from the matrix side of the ovine CV structure (PDB: 6TT7)[2]. Shown in pink is the alpha subunit, in light purple the beta subunit, and the gamma subunit is rendered in dark blue. The red sphere represents the Leu335Pro mutation site in the beta subunit. **B.** Zoom into the mutation site in the beta subunit (red spheres), sites corresponding to the yeast *mgi* mutations are also rendered for reference (yellow spheres).

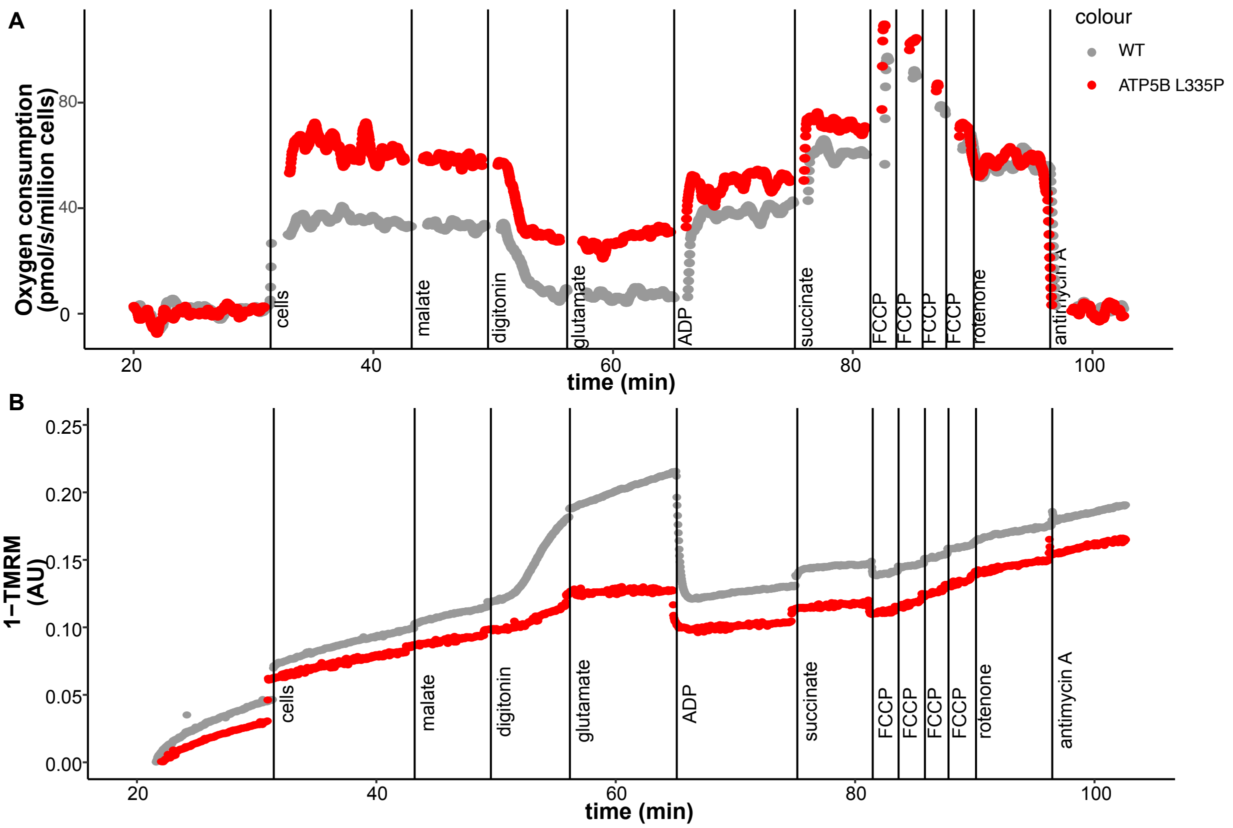

Figure S2. Representative oxygen and TMRM traces for patient fibroblasts. A. Representative oxygen consumption rates in the Oroboros oxygraphy in wild-type cells (grey) or cells with the ATP5B L335P variant (red) in the leak respiratory state following stimulation by complex I substrate glutamate prior to addition of ADP, in the presence of saturating ADP and after complete uncoupling by FCCP. **B.** Simultaneous 1-TMRM fluorescence intensity as an indicator of mitochondrial membrane potential in the same time conditions, where positive change represents polarization and negative change represents depolarization.

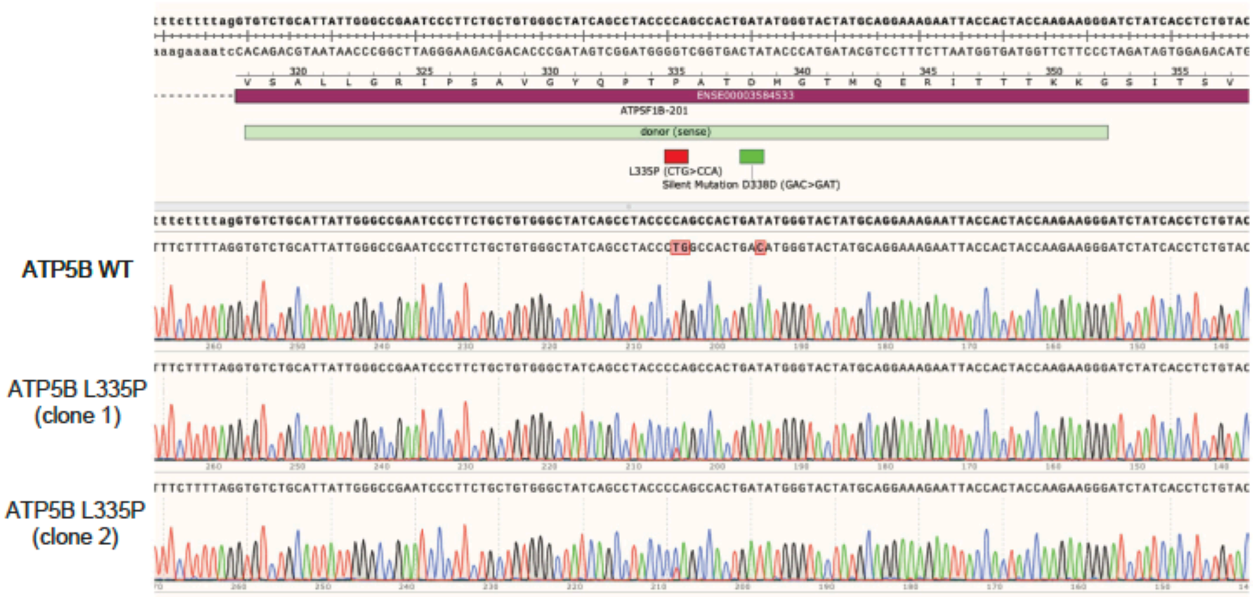

Figure S3: Sequence confirmation of CRISPR engineered isogenic A375 cell lines. The sequence near the edited site of each cell line used in Fig. 3A-C, S4 was confirmed by Sanger sequencing.

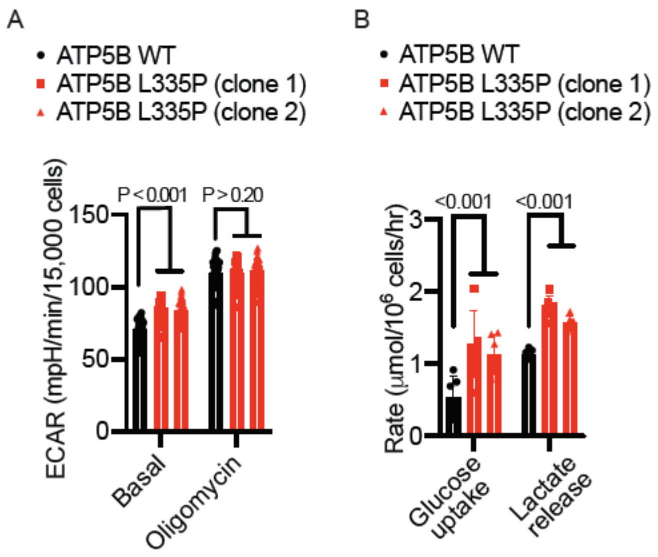

Figure S4: Glycolytic phenotype of the CRISPR engineered isogenic A375 cell lines. A. Seahorse intact cell glycolytic rate measurements under basal and oligomycin (2 μM) conditions. **B.** Glucose consumption and lactate release from metabolite analysis of spent media over an 8 h period.

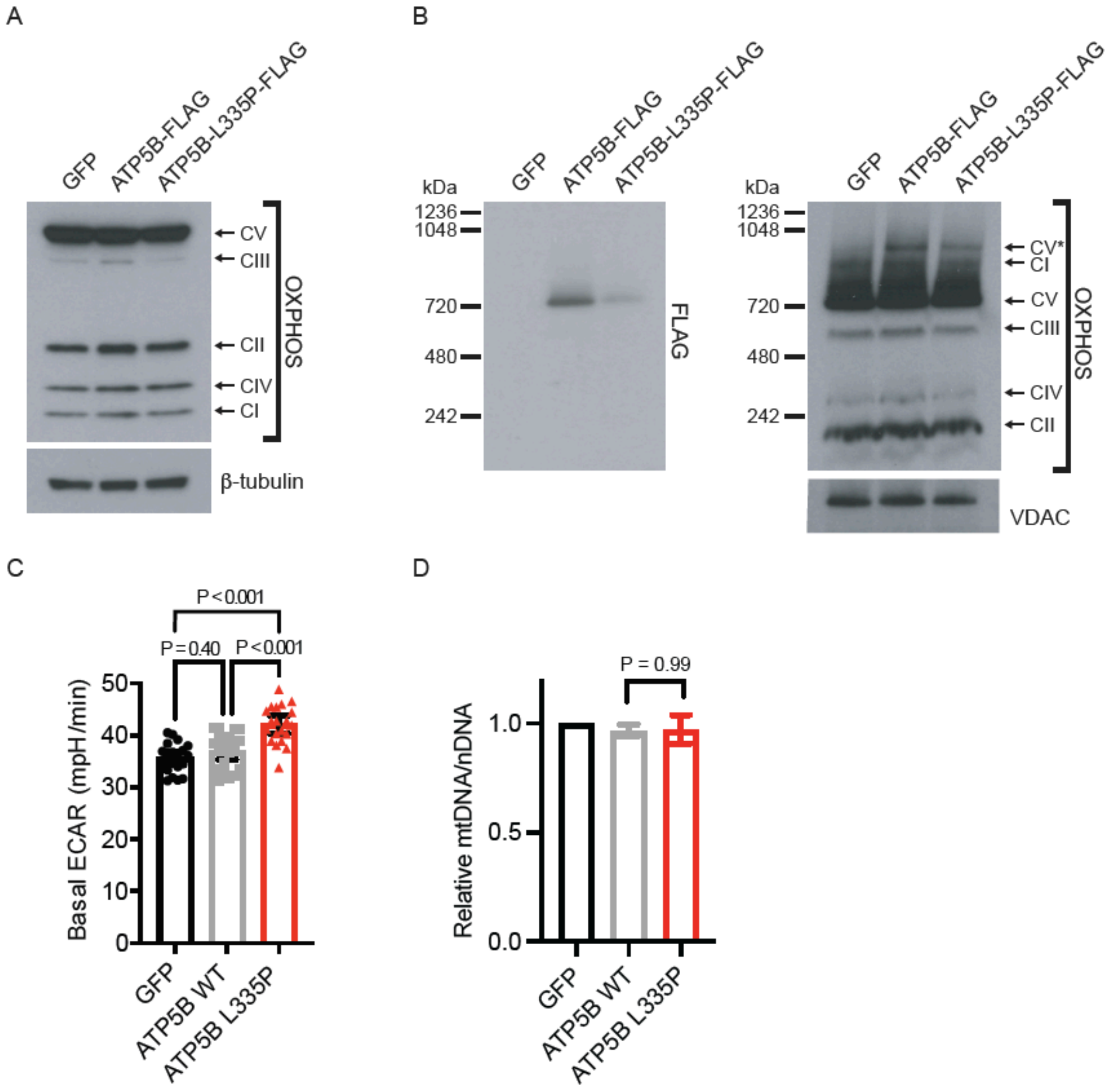

Figure S5: Additional analyses of heterologous expression of the WT and Leu335Pro variant in HeLa cell lines. A. SDS-PAGE and Western blot analysis of OXPHOS complexes of GFP control, FLAG-tagged wild type ATP5B, or the FLAG-tagged Leu335Pro variant in HeLa cells. **B.** (Right panel) Blue Native PAGE and immunoblot analysis of native OXPHOS complexes in HeLa cells. (Left panel) FLAG immunoblot of BN-PAGE shows incorporation of FLAG-tagged WT and Leu335Pro variant ATP5B proteins into mature CV. **C.** Seahorse intact cell glycolytic rate measurements under basal conditions (n=18 biological replicates). **D.** mtDNA copy number measurements by qRT-PCR. For Fig. S5B, asterisk indicates CV-containing supercomplex. ECAR: extracellular acidification rate; For Fig. S5D, error bars represent means ± 95 % CI across the 4 combinations of nDNA / mtDNA probes used in the qRT-PCR analysis from two biological replicates.

Table S1: Metabolic laboratories (Grey rows: Twin A; white rows: Twin B). Red values are outside of the reference range. These laboratory values show elevated ammonia, blood urea nitrogen and branched chain amino acids in both subjects. Triglycerides were also elevated in Twin A. We interpret the results of amino acid profiling, ammonia and urea measurements to suggest that ureagenesis was not impaired (as evidenced by normal-high urea levels); however, that there was excessive nitrogenous waste production (as evidenced by high urea and ammonia), likely related to high protein catabolism rates. This is supported by the high circulating branched chain amino acid levels, which are often seen in times of protein catabolism. Similarly, we speculate that the elevated triglyceride levels reflect mobilization of fatty acids for catabolism. Despite their tachypnea, blood pH, carbon dioxide and bicarbonate levels were normal. ND: Not Determined

	Reference range	3 months	4.5 months	12 months	14 months
**Lactate (mmol/L)**	0.6-2.0	1.8	ND	1.4	1.5
		2.0	ND	1.7	1.7
**Ammonia (µmol/L)**	9-33	**60**	**80**	**52**	**76**
		**102**	**106**	**77**	**101**
**Blood urea nitrogen (mg/dL)**	2-19	**22**	**21**	17	14
		**35**	ND	**23**	**21**
**Triglycerides (mg/dL)**	27-125	**187**	ND	ND	**226**
		119	ND	ND	
**Leucine (µmol/L)**	42-188	81.2	**206**	179	**240**
		143	185	**239**	**222**
**Isoleucine (µmol/L)**	17-106	47.5	**130**	96.9	**132**
		88	**116**	**132**	**111**
**Valine (µmol/L)**	93-321	219	**392**	**395**	**405**
		**330**	**402**	**536**	**451**

Table S2: Developmental milestones achieved at 22 months of age (20 months corrected for gestational age)

	**Twin A**	**Twin B**
**Language development**	Has a single word ("all-done"), points to communicate, follows one-step commands (e.g. "up" or "stand")	Babbles, follows one step commands
**Fine motor development**	Can stack cups, but not yet stack blocks. Can feed himself with his hands, but not use utensils	Can stack cups, but not yet stack blocks. Can feed himself with a spoon and drink from a straw cup
**Gross motor development**	Stands independently. Able to take a few steps.	Stands with support

Table S3: cDNA, primer, and guide RNA sequences

cDNA	Sequence	Notes
ATP5B-FLAG	TCGACCTGCTAGCATGTTGG<br>GGTTTGTGGGTCGGGTGGCC<br>GCTGCTCCGGCCTCCGGGGC<br>CTTGCGGAGACTCACCCCTT<br>CAGCGTCGCTGCCCCCAGCT<br>CAGCTCTTACTGCGGGCCGC<br>TCCGACGGCGGTCCATCCTG<br>TCAGGGACTATGCGGCGCAA<br>ACATCTCCTTCGCCAAAAGC<br>AGGCGCCGCCACCGGGCGCA<br>TCGTGGCGGTCATTGGCGCA<br>GTGGTGGACGTCCAGTTTGA<br>TGAGGGACTACCACCAATTC<br>TAAATGCCCTGGAAGTGCAA<br>GGCAGGGAGACCAGACTGGT<br>TTTGGAGGTGGCCCAGCATT<br>TGGGTGAGAGCACAGTAAGG<br>ACTATTGCTATGGATGGTAC<br>AGAAGGCTTGGTTAGAGGCC<br>AGAAAGTACTGGATTCTGGT<br>GCACCAATCAAAATTCCTGT<br>TGGTCCTGAGACTTTGGGCA<br>GAATCATGAATGTCATTGGA<br>GAACCTATTGATGAAAGAGG<br>TCCCATCAAAACCAAACAAT<br>TTGCTCCCATTCATGCTGAGG<br>CTCCAGAGTTCATGGAAATG<br>AGTGTTGAGCAGGAAATTCT<br>GGTGACTGGTATCAAGGTTG<br>TCGATCTGCTTGCTCCCTATG<br>CCAAGGGTGGCAAAATTGGG<br>CTTTTTGGTGGTGCTGGAGTT<br>GGCAAGACTGTACTGATCAT<br>GGAGTTAATCAACAATGTCG<br>CCAAAGCCCATGGTGGTTAC<br>TCTGTGTTTGCTGGTGTTGGT<br>GAGAGGACCCGTGAAGGCA<br>ATGATTTATACCATGAAATG<br>ATTGAATCTGGTGTTATCAA<br>CTTAAAAGATGCCACCTCTA<br>AGGTAGCGCTGGTATATGGT<br>CAAATGAATGAACCACCTGG<br>TGCTCGTGCCCGCGTAGCTCT	Internal restriction sites modified to facilitate cloning, with no change to wild type protein sequence

	GACTGGGCTGACTGTGGCTG AATACTTCAGAGACCAAGAA GGTCAAGATGTACTGCTATT TATTGATAACATCTTTCGCTT CACCCAGGCTGGTTCAGAGG TGTCTGCATTATTGGGCCGA ATCCCTTCTGCTGTGGGCTAT CAGCCTACCCTGGCCACTGA CATGGGTACTATGCAGGAAA GAATTACCACTACCAAGAAG GGATCTATCACCTCTGTACA GGCTATCTATGTGCCTGCTG ATGACTTGACTGACCCTGCC CCTGCTACTACGTTTGCCCAT TTGGATGCTACCACTGTACT GTCGCGTGCCATTGCTGAGC TGGGCATCTATCCAGCTGTG GATCCTCTAGACTCCACCTCT CGTATCATGGATCCCAACAT TGTTGGCAGTGAGCATTACG ATGTTGCCCGTGGGGTGCAA AAGATCCTGCAGGACTACAA ATCCCTCCAGGATATCATTG CCATCCTGGGTATGGATGAA CTTTCTGAGGAAGACAAGTT GACCGTGTCCCGTGCACGGA AAATACAGCGTTTCTTGTCTC AGCCATTCCAGGTTGCTGAG GTCTTCACAGGTCATATGGG GAAGCTGGTTCCCCTGAAGG AGACCATCAAAGGATTCCAG CAGATTTTGGCAGGTGAATA TGACCATCTCCCAGAACAGG CCTTCTATATGGTGGGACCC ATTGAAGAAGCTGTGGCAAA AGCTGATAAGCTGGCTGAAG AGCATTCATCGGGTGGATCT GGTGGATCTGGTGGATCTGA TTACAAGGATGACGATGACA AGTAAGAATTCATCGACCA	
ATP5B-L335P-FLAG	TCGACCTGCTAGCATGTTGG GGTTTGTGGGTCGGGTGGCC GCTGCTCCGGCCTCCGGGGC CTTGCGGAGACTCACCCCTT CAGCGTCGCTGCCCCCAGCT	Internal restriction sites modified to facilitate cloning, with no change to variant codon or protein sequence

	CAGCTCTTACTGCGGGCCGC<br>TCCGACGGCGGTCCATCCTG<br>TCAGGGACTATGCGGCGCAA<br>ACATCTCCTTCGCCAAAAGC<br>AGGCGCCGCCACCGGGCGCA<br>TCGTGGCGGTCATTGGCGCA<br>GTGGTGGACGTCCAGTTTGA<br>TGAGGGACTACCACCAATTC<br>TAAATGCCCTGGAAGTGCAA<br>GGCAGGGAGACCAGACTGGT<br>TTTGGAGGTGGCCCAGCATT<br>TGGGTGAGAGCACAGTAAGG<br>ACTATTGCTATGGATGGTAC<br>AGAAGGCTTGGTTAGAGGCC<br>AGAAAGTACTGGATTCTGGT<br>GCACCAATCAAAATTCCTGT<br>TGGTCCTGAGACTTTGGGCA<br>GAATCATGAATGTCATTGGA<br>GAACCTATTGATGAAAGAGG<br>TCCCATCAAAACCAAACAAT<br>TTGCTCCCATTCATGCTGAGG<br>CTCCAGAGTTCATGGAAATG<br>AGTGTTGAGCAGGAAATTCT<br>GGTGACTGGTATCAAGGTTG<br>TCGATCTGCTTGCTCCCTATG<br>CCAAGGGTGGCAAAATTGGG<br>CTTTTTGGTGGTGCTGGAGTT<br>GGCAAGACTGTACTGATCAT<br>GGAGTTAATCAACAATGTCG<br>CCAAAGCCCATGGTGGTTAC<br>TCTGTGTTTGCTGGTGTTGGT<br>GAGAGGACCCGTGAAGGCA<br>ATGATTTATACCATGAAATG<br>ATTGAATCTGGTGTTATCAA<br>CTTAAAAGATGCCACCTCTA<br>AGGTAGCGCTGGTATATGGT<br>CAAATGAATGAACCACCTGG<br>TGCTCGTGCCCGCGTAGCTCT<br>GACTGGGCTGACTGTGGCTG<br>AATACTTCAGAGACCAAGAA<br>GGTCAAGATGTACTGCTATT<br>TATTGATAACATCTTTCGCTT<br>CACCCAGGCTGGTTCAGAGG<br>TGTCTGCATTATTGGGCCGA<br>ATCCCTTCTGCTGTGGGCTAT<br>CAGCCTACCCCGGCCACTGA	

	CATGGGTACTATGCAGGAAA<br>GAATTACCACTACCAAGAAG<br>GGATCTATCACCTCTGTACA<br>GGCTATCTATGTGCCTGCTG<br>ATGACTTGACTGACCCTGCC<br>CCTGCTACTACGTTTGCCCAT<br>TTGGATGCTACCACTGTACT<br>GTCGCGTGCCATTGCTGAGC<br>TGGGCATCTATCCAGCTGTG<br>GATCCTCTAGACTCCACCTCT<br>CGTATCATGGATCCCAACAT<br>TGTTGGCAGTGAGCATTACG<br>ATGTTGCCCGTGGGGTGCAA<br>AAGATCCTGCAGGACTACAA<br>ATCCCTCCAGGATATCATTG<br>CCATCCTGGGTATGGATGAA<br>CTTTCTGAGGAAGACAAGTT<br>GACCGTGTCCCGTGCACGGA<br>AAATACAGCGTTTCTTGTCTC<br>AGCCATTCCAGGTTGCTGAG<br>GTCTTCACAGGTCATATGGG<br>GAAGCTGGTTCCCCTGAAGG<br>AGACCATCAAAGGATTCCAG<br>CAGATTTTGGCAGGTGAATA<br>TGACCATCTCCCAGAACAGG<br>CCTTCTATATGGTGGGACCC<br>ATTGAAGAAGCTGTGGCAAA<br>AGCTGATAAGCTGGCTGAAG<br>AGCATTCATCGGGTGGATCT<br>GGTGGATCTGGTGGATCTGA<br>TTACAAGGATGACGATGACA<br>AGTAAGAATTCATCGACCA	
pLJM-reverse sequencing primer	GACGTGAAGAATGTGCGAGA	Reverse sequencing primer for pLJM1 and pLYS1
SR5F-Hs-mttRNALeu	CACCCAAGAACAGGGTTTGT	Primer for mtDNA copy number assay
SR5R-Hs-mttRNALeu	TGGCCATGGGTATGTTGTTA	Primer for mtDNA copy number assay
SR6F-Hs-mtND6	CCACACCGCTAACAATCAAT<br>AC	Primer for mtDNA copy number assay
SR6R-Hs-mtND6	GTTTCTGTTGAGTGTGGGTTT<br>AG	Primer for mtDNA copy number assay
SR7F-Hs-B2M	GCTGGGTAGCTCTAAACAAT<br>GTATTCA	Primer for mtDNA copy number assay

SR7R-Hs-B2M	CCATGTACTAACAAATGTCTAAAATGGT	Primer for mtDNA copy number assay
SR8F-Hs-45SrRNA	CTTGTCTCAAAGATTAAGCCATGC	Primer for mtDNA copy number assay
SR8R-Hs-45SrRNA	GAGCGACCAAAGGAACCATAA	Primer for mtDNA copy number assay
Guide RNA sequence	UAGUACCCAUGUCAGUGGCC	Guide RNA for generating ATP5B Leu335Pro knock-in
Donor sequence	CCCTTCTTGGTAGTGGTAATTCTTTCCTGCATAGTACCCATATCAGTGGCTGGGGTAGGCTGATAGCCCACAGCAGAAGGGATTCGGCCCAATAATGCAGACA	Donor sequence for generating ATP5B Leu335Pro knock-in

Table S4: Classification of pathogenicity by American College of Medical Genetics and Genomics/Association of Molecular Pathology guidelines

**Criterion** [3]	**Applicability to NM_001686.3:c.1004 T>C**
**Strong Criteria for Pathogenicity**	
PS1 Same amino acid change as a previously established pathogenic variant	No
PS2 De novo (both maternity and paternity confirmed) in a patient with the disease and no family history	*De novo* with confirmed parentage in affected monozygotic twins
PS3 Well-established in vitro or in vivo functional studies supportive of a damaging effect on the gene or gene product	Oxygen consumption and membrane potential studies confirm predicted uncoupling effect; both CRISPR-based knock-in & heterologous expression in engineered cells recapitulate the biochemical findings
PS4 The prevalence of the variant in affected individuals is significantly increased compared with the prevalence in controls	N/A (this paper reports a novel variant, i.e., the only two known affected individuals harbor this variant, which has never before been reported)
**Moderate Criteria for Pathogenicity**	
PM1 Located in a mutational hot spot and/or critical and well-established functional domain (e.g., active site of an enzyme) without benign variation	Impacts a highly conserved residue in the critical hydrophobic sleeve. Nearby mutations in the yeast enzyme are known to cause uncoupling. Benign variation is unknown in this region and uncommon in the gene (*ATP5F1B* probability of being loss-of-function intolerant pLI = 0.98; with far fewer missense (150 observed) and loss of function variants (2 observations) than expected (306 and 20.4, respectively)
PM2 Absent from controls	Absent from >200,000 control alleles in gnomAD
PM3 For recessive disorders, detected in trans with a pathogenic variant	N/A
PM4 Protein length changes as a result of in-frame deletions/insertions in a nonrepeat region or stop-loss variants	No
PM5 Novel missense change at an amino acid residue where a different missense change determined to be pathogenic has been seen before	No
PM6 Assumed de novo, but without confirmation of paternity and maternity	N/A (meets criteria for PS2)
**Variants are classified pathogenic with >= 2 Strong criteria**	2 Strong criteria (PS2, PS3); 2 moderate criteria (PM1, PM2)

N/A: not applicable

References

1. Doerrier C, Garcia-Souza LF, Krumschnabel G, Wohlfarter Y, Mészáros AT, Gnaiger E. High-resolution fluorespirometry and oxphos protocols for human cells, permeabilized fibers from small biopsies of muscle, and isolated mitochondria. In: Methods in Molecular Biology. Humana Press Inc.; 2018. p. 31–70.
2. Pinke G, Zhou L, Sazanov LA. Cryo-EM structure of the entire mammalian F-type ATP synthase. Nat Struct Mol Biol [Internet] 2020;27(11):1077–85. Available from: http://www.ncbi.nlm.nih.gov/pubmed/32929284
3. Richards S, Aziz N, Bale S, et al. Standards and guidelines for the interpretation of sequence variants: a joint consensus recommendation of the American College of Medical Genetics and Genomics and the Association for Molecular Pathology. Genetics in Medicine [Internet] 2015;17(5):405–23. Available from: http://www.nature.com/articles/gim201530
